# Effects of chronic low dose rotenone treatment on human microglial cells

**DOI:** 10.1186/1750-1326-4-55

**Published:** 2009-12-31

**Authors:** Shamim B Shaikh, Louise FB Nicholson

**Affiliations:** 1 Department of Anatomy with Radiology and The Centre for Brain Research, Faculty of Medical and Health Sciences, University of Auckland, Private Bag 92019, Auckland, New Zealand

## Abstract

**Background:**

Exposure to toxins/chemicals is considered to be a significant risk factor in the pathogenesis of Parkinson's disease (PD); one putative chemical is the naturally occurring herbicide rotenone that is now used widely in establishing PD models. We, and others, have shown that chronic low dose rotenone treatment induces excessive accumulation of Reactive Oxygen Species (ROS), inclusion body formation and apoptosis in dopaminergic neurons of animal and human origin. Some studies have also suggested that microglia enhance the rotenone induced neurotoxicity. While the effects of rotenone on neurons are well established, there is little or no information available on the effect of rotenone on microglial cells, and especially cells of human origin. The aim of the present study was to investigate the effects of chronic low dose rotenone treatment on human microglial CHME-5 cells.

**Methods:**

We have shown previously that rotenone induced inclusion body formation in human dopaminergic SH-SY5Y cells and therefore used these cells as a control for inclusion body formation in this study. SH-SY5Y and CHME-5 cells were treated with 5 nM rotenone for four weeks. At the end of week 4, both cell types were analysed for the presence of inclusion bodies, superoxide dismutases and cell activation (only in CHME-5 cells) using Haematoxylin and Eosin staining, immunocytochemical and western blotting methods. Levels of active caspases and ROS (both extra and intra cellular) were measured using biochemical methods.

**Conclusion:**

The results suggest that chronic low dose rotenone treatment activates human microglia (cell line) in a manner similar to microglia of animal origin as shown by others. However human microglia release excessive amounts of ROS extracellularly, do not show excessive amounts of intracellular ROS and active caspases and most importantly do not show any protein aggregation or inclusion body formation. Human microglia appear to be resistant to rotenone (chronic, low dose) induced damage.

## Background

Parkinson's disease (PD) is the most prevalent neurodegenerative movement disorder [1]; genetic mutations are found in rare cases of familial PD, however more than 90% of cases are sporadic without known cause [2]. The role of microglia, the resident immune cells of the Central Nervous System (CNS), has rapidly gained attention from researchers interested in understanding the pathogenesis of PD. Chronic microglial activation, subsequent neuroinflammation and accumulation of Reactive Oxygen Species (ROS) are now accepted as significant contributors in the disease progression [3,4]. The importance of the role played by microglia in inducing neuroinflammation in PD is highlighted by the fact that the substantia nigra, a region that shows extensive degeneration of dopaminergic neurons in PD, is also the region most densely populated with microglial cells [5,6]. Dopaminergic neurons also show a unique vulnerability to the factors released by activated microglia. It is therefore important to fully understand the interaction between microglia and neurons and the precise mechanisms by which microglia influence dopaminergic neuronal cell loss through the use of *in-vitro *and *in-vivo *models of PD.

Neurodegenerative diseases are difficult to model. A number of epidemiological studies have identified a strong correlation between exposure to pesticides and increased incidence of PD [7-11], suggesting pesticides as the accelerators/promoters of PD. Some of the pesticides in question were found to inhibit complex I enzyme activity [8,12]. Based on these experimental and clinical findings, PD was the first neurological disease to be modelled. Chemicals such as reserpine, methamphetamine, 6-hydroxydopamine, 1-methyl-4-phenyl-1,2,3,6-tetrahydroxypyridine (MPTP), paraquat and rotenone have all been used to make PD models [13,14]. Studies by Betarbet and colleagues [12,15,16], reported that chronic treatment with the herbicide rotenone led to selective destruction of nigrostriatal dopaminergic neurons, formation of cytoplasmic inclusions in nigral neurons and induction of hypokinesia and rigidity in rats. These studies have drawn a lot of attention highlighting rotenone's potential use as a model for toxin-induced Parkinsonism.

Rotenone is commonly used as a natural pesticide, an insecticide and to kill 'nuisance' fish in lakes. Rotenone is a lipophilic compound and can easily cross the blood-brain barrier [13,14]. Though chronic exposure to low doses of rotenone results in uniform inhibition of complex I throughout the brain, rotenone has been shown to induce selective degeneration of the nigrostriatal pathway [12]. Although some researchers [17,18] have reported non-specific central nervous system and systemic toxicity in rats upon rotenone treatment, recently Cannon et al., (2009) [2] have reproduced consistently many features of PD in rats following chronic intraparitoneal administration of rotenone. Features include systemic mitochondrial impairment, oxidative damage, microglial activation, selective nigrostriatal dopaminergic degeneration, L-Dopa responsive motor deficit, gastrointestinal deficit and α-synuclein and ubiquitin positive lewy body-like inclusion bodies within TH-positive neurons. Rotenone has also been used successfully to reproduce PD characteristics in *in-vitro *slice culture models [19], primary cultures [20] and recently we have shown accumulation of α-synuclein positive inclusions within four weeks of chronic low dose treatment of SH-SY5Y dopaminergic cells of human origin [21].

Microglial activation and neuroinflammation have been identified as important contributors in human PD pathogenesis, and several rotenone models have demonstrated microglial activation in the striatum of the treated animals [2,12]. Recent studies have reported that microglia contribute significantly in mediating and potentiating the neurodegenerative effects of rotenone [22,23]. There is however, very little or no information available on the direct effects of rotenone on microglial cells. Some authors have reported microglial activation upon rotenone treatment in the microglia of rodent origin [2,12]. There are however some important differences between human and rodent microglia in the way they release superoxide in response to the same activating agents. Furthermore, and in contrast to rodent microglia, human microglia are thought to produce very little or no inducible nitric oxide synthase (iNOS) [24,25]. In this study, we report the direct effects of chronic low dose rotenone treatment on human microglial cells.

## Results

### Chronic low dose rotenone treatment does not induce intracellular inclusion formation in human microglial cells

**Figure 1:**
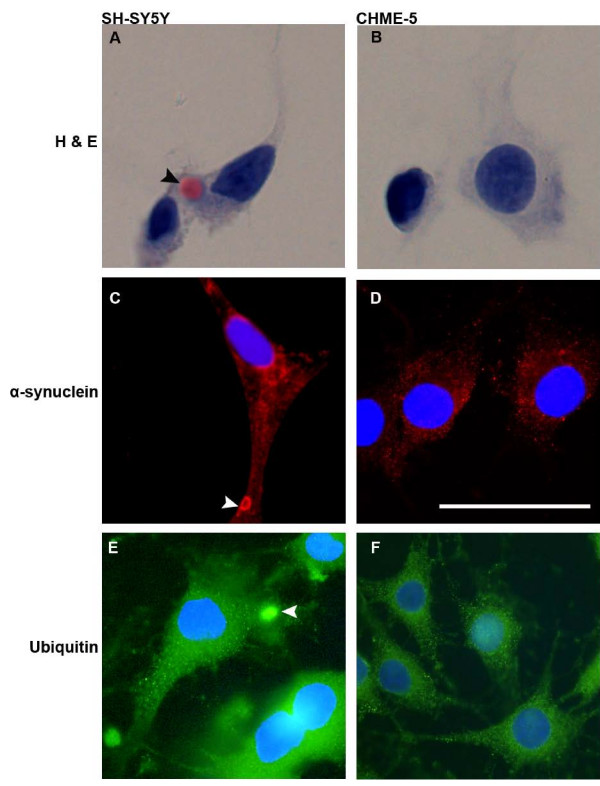
**Detection of inclusion bodies**. Representative photomicrographs showing H & E staining (A and B), α-synuclein staining (C & D) and ubiquitin staining (E and F) in the two cell types (SH-SY5Y and CHME-5) collected at the end of 4 week's of rotenone (5 nm) treatment. SH-SY5Y cells showed eosinophilic (A, arrow head), α-synuclein positive (C, arrow head) and ubiquitin positive (E, arrow head) inclusion bodies. No inclusion bodies were seen in human microglial CHME-5 cells (B, D and F). Results shown are representative of three independent experiments. Scale bar: 50 μm.

We have reported previously the appearance of cytoplasmic α-synuclein positive inclusion bodies in human dopaminergic SH-SY5Y cells [21]. In the present study we investigated the effects of chronic low dose rotenone treatment on the human microglial cells, using SH-SY5Y cells as a control for the induction of inclusion bodies. CHME-5 and SH-SY5Y cells were treated in the same way and examined for the presence of inclusion bodies at the end of four weeks using H & E staining and immunocytochemical staining for α-synuclein and ubiquitin. As reported earlier by us [21], the inclusion bodies were seen in SH-SY5Y cells within 4 weeks of rotenone treatment; these inclusion bodies were eosinophilic and positive for both α-synuclein and ubiquitin (Figure 1A, C and 1E). Interestingly no inclusion bodies were seen in the human microglial CHME-5 cells (Figure 1B, D and 1F) indicating that the CHME-5 cells are more resistant to inclusion formation and to rotenone induced damage. No inclusion bodies were seen in the control group of both cell types (data not shown).

### Rotenone treatment activates microglia

**Figure 2:**
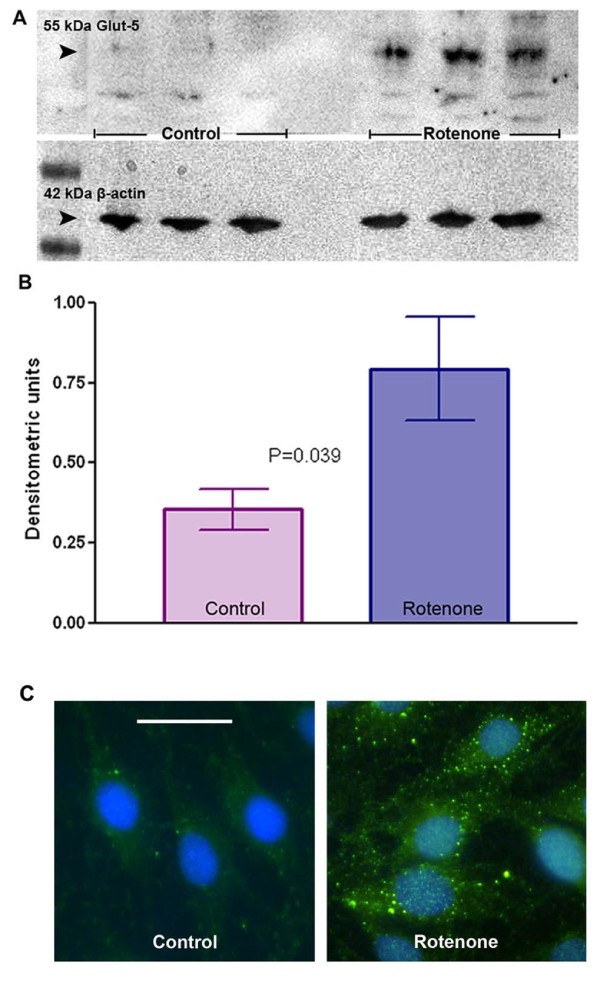
**Glut-5 levels in microglial CHME-5 cells**. **Figure 2A**: Representative immunoblot showing the levels of Glut-5 protein (55 kDa, first row) in the cell lysates collected at the end of week 4 from the control and rotenone (5 nm) treated CHME-5 cells. β-actin (42 kDa, second row) was used as an internal loading control. **Figure 2B**: Histogram showing relative semi-quantitation of Glut-5 levels. Glut-5 levels were normalised against the β-actin levels in each well and expressed in densitometric units. P (p = 0.039) value indicates a significant difference in Glut-5 levels between control and rotenone treated groups. Each column represents the mean and standard deviation from three independent experiments. **Figure 2C**: Representative photomicrographs showing immunostaining of CR3/43 (HLA DR/DP/DQ) in control and rotenone treated CHME-5 cells at the end of week 4 of the treatment period. CR3/43 staining is dramatically increased in rotenone treated cells as visualised by the green fluorescence. Scale bar: 50 μm.

Glut-5 band (55 kDa) was not detected in untreated control CHME-5 cells in the amount (15 μg) of total protein loaded in this study (Figure 2A). However rotenone treated CHME-5 cells showed a significant increase in the levels of Glut-5 proteins at the end of week four (Figure 2A and 2B). Glut-5 is a glucose transporter, specifically expressed in resting and reactive microglia with its expression increased upon activation [26,27]. Activation of microglia upon rotenone treatment was also confirmed by CR3/43 immunostaining; CR3/43 is a marker for activated microglia. CR3/43 immunostaining per cell was increased dramatically in rotenone treated cells compared to controls (Figure 2C). A relative 2.27 fold increase in Glut-5 levels and a dramatic increase in intracellular CR3/43 immunostaining in rotenone treated cells compared to controls suggest increased microglial activation in the rotenone treated group.

### Microglia do not accumulate intracellular ROS upon treatment with rotenone

**Figure 3:**
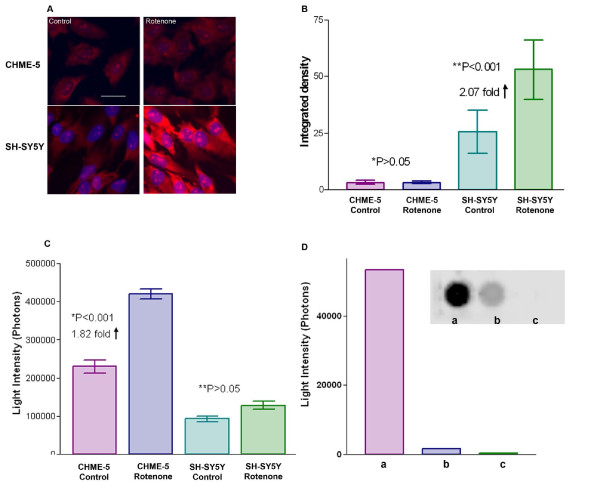
**Detection of Reactive Oxygen Species (ROS)**. **Figure 3A**: Detection of ROS using DHE fluorescent dye in CHME-5 and SH-SY5Y cells from the two treatment groups (DMSO control and 5 nm rotenone) at the end of week 4. Hoechst 33258 stain (blue) was used to identify the nucleus of cells. The fluorescent intensity of the red dots in the nucleus indicates the relative levels of ROS present in the cell. Scale bar: 20 μm. **Figure 3B**: Histogram showing the levels of ROS in each of the treatment groups in both cell types (CHME-5 and SH-SY5Y) at the end of week 4. The levels are expressed in densitometric units. P (*p > 0.05) value indicates that there was no significant difference in ROS levels between the control and rotenone treated CHME-5 cells. However rotenone treated SH-SY5Y cells showed a significant (**P < 0.001) two fold increase in the intracellular ROS levels compared to the untreated controls. Each column represents the mean and standard deviation from three independent experiments **Figure 3C**: Histogram representing the extracellular levels of ROS using the Acridan Lumigen PS-3 assay in the medium collected from both treatment groups (DMSO control and 5 nm rotenone) and both cell types (CHME-5 and SH-SY5Y) at the end of week 4. The levels are expressed in light intensity units. CHME-5 cells released significantly higher (*p < 0.001) levels of ROS in the medium compared to controls, while the extracellular release of ROS in the medium from rotenone treated and untreated SH-SY5Y cells was not significantly different (**P > 0.05). Each column represents the mean and standard deviation from three independent experiments. **Figure 3D**: Image showing the effects of antioxidants on chemiluminescence in the Acridan Lumigen PS-3 assay. Chemiluminescent signals were captured using Fujifilm Luminescent Image Analyzer (Dots) and light intensity units were recorded using the BioTek Synergy 2 plate reader (bar graph). a: culture medium from CHME-5 cells treated with 5 nm rotenone for 4 weeks, b: culture medium (as in a) incubated with 100 μM ascorbic acid (vitamin C) for 5 minutes, c: culture medium (as in a) incubated with NuPAGE antioxidant (1:500 diluted). The dramatic reduction of chemiluminescence in the media incubated with antioxidants indicates that the chemiluminescence in Acridan PS-3 assay is ROS specific.

Reactive oxygen Species (ROS) were measured using the DHE assay for intracellular and the Acridan Lumigen PS-3 assay for extracellular ROS in both cell types (CHME-5 and SH-SY5Y) at the end of 4 week's rotenone treatment. The amount of ROS accumulated within cells was not significantly different in the CHME-5 cells treated with low dose rotenone compared to untreated controls (Figure 3A and 3B), however, as reported previously by us [21], rotenone treated SH-SY5Y cells showed a 2 fold significant increase in intracellular ROS compared to the untreated controls (Figure 3A and 3B). Interestingly, the CHME-5 cells release excessive amounts of ROS extracellularly, as detected in the culture medium, while the levels of extracellular ROS in the culture media from rotenone treated and untreated SH-SY5Y cells were approximately the same (Figure 3C). These results suggest that, despite microglial activation upon rotenone treatment, ROS are not accumulated in excessive amounts within microglial cells. These cells release large amounts of ROS extracellularly into the culture media. In contrast, rotenone treated SH-SY5Y cells accumulate excessive ROS (generated as a result of rotenone treatment) within cells.

Incubation of the culture media from rotenone treated cells with 100 μM Ascorbic acid (Vitamin C) reduced the chemiluminescence dramatically whereas incubation of the culture media from rotenone treated CHME-5 cells with NuPAGE antioxidant completely inhibited the chemiluninescence in the Acridan Ps-3 assay (Figure 3D) suggesting that the chemiluminescence generated in the Acridan PS-3 assay was specifically due to the presence of ROS in the culture media. Vitamin C incubation did not completely inhibit the chemiluminescence, suggesting that the ROS other than superoxide were also present. Superoxide appears to be the main ROS present as indicted by the dramatic reduction in the light intensity after incubation with Vitamin C (Figure 3D).

### Chronic low dose rotenone treatment does not induce apoptosis in human microglia

**Figure 4:**
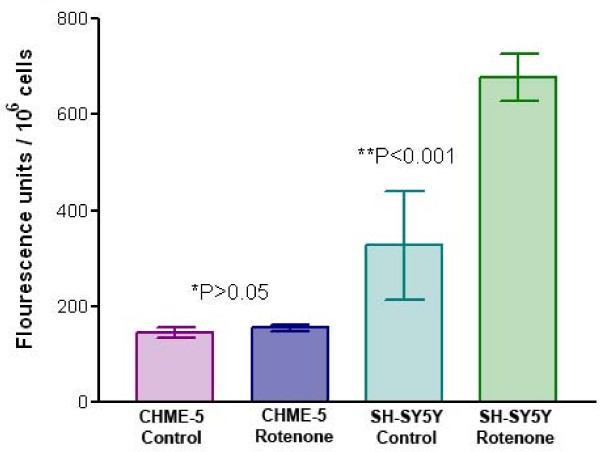
**Detection of active caspases using FLICA assay**. Histogram showing the levels of active caspases in each of the treatment groups from the two cell types (CHME-5 and SH-SY5Y) at the end of week 4. The levels are expressed in fluorescence units/10^6 ^cells. P (*P > 0.05) value indicates that there was no significant difference in the level of active caspases between the rotenone treated and untreated CHME-5 cells. Rotenone treated SH-SY5Y cells showed significantly increased (**P < 0.001) levels of active caspases (fluorescence units) compared to untreated control cells. Each column represents the mean and standard deviation from three independent experiments.

FLICA assay was performed in both cell types at the end of the four week's rotenone treatment. SH-SY5Y cells were used as a positive control for apoptosis and to confirm FLICA assay's specificity. FLICA reagent SR-VAD-FMK binds to all active caspases (caspase-1, -3, -4, -5, -6, -7, -8 and -9), the cells in the early stages of apoptosis emit red fluorescence and the intensity of fluorescent light increases with the advancement in the stage of apoptosis. Non-apoptotic cells mainly remain unstained (information provided by the Immunocytochemistry Technologies, LLC, Bloomington, MN). We did not separate the cell populations exhibiting different intensities of fluorescence to analyse the percentage of cells in early or late stage of apoptosis. The measurement of overall fluorescence intensity in cells, using fluorometry, suggests that low dose treatment with rotenone does not induce accelerated cell death signals in human microglial cells; at the end of week four, there was no significant difference in the levels of active caspases between rotenone treated CHME-5 cells and the controls (Figure 4). The microscopic observations suggested that cells in both treatment groups were fluorescing red with similar intensity (data not shown) indicating activation of some caspases at reduced levels. SH-SY5Y cells were used as a positive control for apoptosis. The significant increase in the levels of active caspases in rotenone treated SH-SY5Y cells compared to controls and rotenone treated CHME-5 cells indicates that the low dose rotenone treatment does not induce apoptosis in CHME-5 cells whereas SH-SY5Y cells are more prone to apoptosis upon rotenone treatment.

### SOD1 but not SOD2 increases significantly upon rotenone treatment

**Figure 5:**
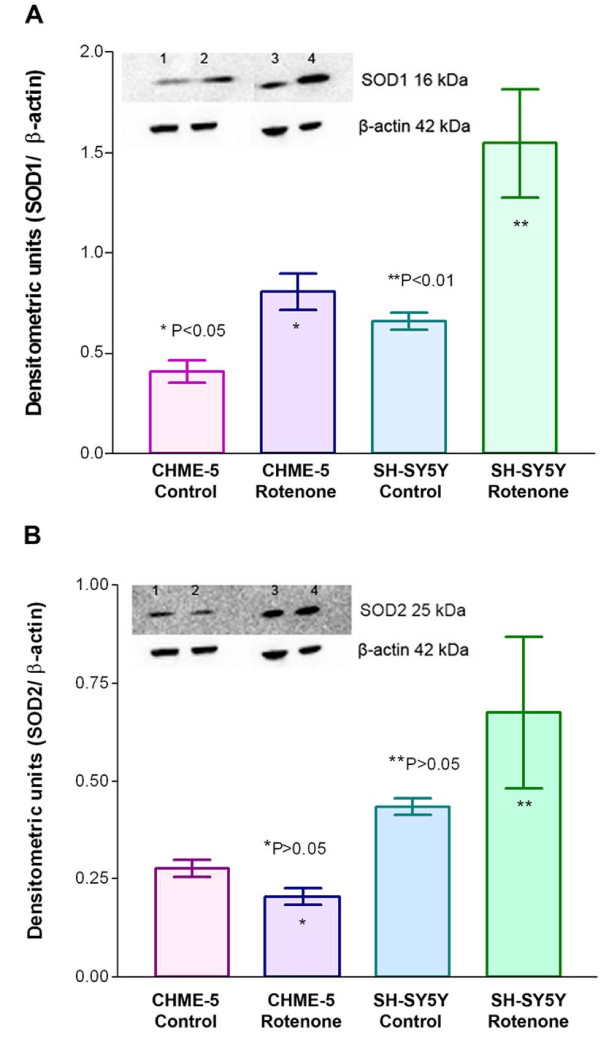
**Superoxide dismutase (SOD) levels in both CHME-5 and SH-SY5Y cells**. **Figure 5A**: Representative immunoblot and histogram showing levels of SOD1 in the two types of cells (CHME-5 and SH-SY5Y) collected from two treatment groups (DMSO control and rotenone treated). CHME-5 cells showed a significant (p < 0.05) two fold increase in SOD1 levels, whereas SH-SY5Y cells showed a more significant 2.4 fold increase (p < 0.01) in SOD1 levels after 4 weeks of rotenone treatment. The base levels of SOD1 appeared to be higher in SH-SY5Y cells compared to CHME-5 cells. **Figure 5B: **Representative immunoblot and histogram showing levels of SOD2 in the two types of cells (CHME-5 and SH-SY5Y) collected from two treatment groups (DMSO control and rotenone treated). CHME-5 cells did not show any change in SOD2 levels (p > 0.05), whereas SH-SY5Y cells showed a 1.5 fold increase in SOD2 levels after 4 weeks of rotenone treatment, although this increase was not significant (p > 0.05). The base levels of SOD2 appeared to be higher in SH-SY5Y cells compared to CHME-5 cells. 1: CHME-5 control; 2: CHME-5 rotenone; 3: SH-SY5Y control; 4: SH-SY5Y rotenone. Each column represents the mean and standard deviation from three independent experiments.

The main ROS released as a result of mitochondrial enzyme inhibition is superoxide [22,23,28]. We therefore analysed the levels of SODs (1 and 2) to determine if microglia are protected as a result of excessive SOD levels. In our previous study we reported accumulation of excessive amounts of ROS in SH-SY5Y cells [21]. SH-SY5Y cells were therefore used as controls in most of these experiments. Interestingly, the basal level of both cytoplasmic SOD1 and mitochondrial SOD2 were higher (1.6 fold) in dopaminergic SH-SY5Y cells compared to microglial CHME-5 cells (Figure 5A and 5B respectively). SOD1 levels increased significantly in microglial cells upon rotenone treatment at the end of 4 weeks to around 2 fold (Figure 5A) and no change was observed in SOD2 levels (Figure 5B). The increase in SOD 1 level was more dramatic (2.4 fold) in SH-SY5Y cells upon rotenone treatment for 4 weeks (Figure 5A). These cells also showed around 1.5 fold increase in SOD2 levels compared to controls, however this increase was not significant (Figure 5B).

## Discussion

Exposure to environmental toxins is considered to be a significant contributor to the pathogenesis of PD. Since the first report by Betarbet et al. in 2000 [12] that chronic treatment with the herbicide rotenone leads to selective destruction of nigrostriatal dopaminergic neurons in rodents, rotenone has been used widely for establishing *in-vivo *and *in-vitro *models of PD. We and others have also shown that chronic, low dose treatment of human dopaminergic neuroblastoma SH-SY5Y cells induces α-synuclein positive intraneuronal inclusion bodies [15,21]. Rotenone-induced dopaminergic neurodegeneration is attributed to both the mitochondrial impairment within neurons and enhancement of microglial activation [22]. Using transgenic mice and primary mesencephalic cultures, researchers have shown that activated microglia greatly enhance the rotenone induced neurotoxicity through the production of pro-inflammatory cytokines and NADPH oxidase derived superoxide [22,23]. The effect of rotenone on dopaminergic neurons and the contribution of microglia to neuronal death have been extensively studied. There is however little information available on the effect of rotenone treatment on microglia although some researchers have reported microglial activation upon exposure to rotenone in microglia of animal origin using rodent *in-vivo *models and primary mesencephalic cultures [2,12,29]. There are some differences between microglia of animal and human origin especially in the way they produce the superoxide anion and iNOS (inducible nitric oxide synthase) in response to the same activating agent [24,25]. Therefore, it is important to determine the effect of rotenone on microglia of human origin. Due to limited availability of human tissue, low yield of pure human microglial cultures and ethical issues on the use of human tissue, we used the transformed human microglial cell line (CHME-5). To the best of our knowledge this is the first study investigating the effect of chronic low dose treatment of rotenone on human microglial (CHME-5) cells.

In the present study we have shown that human microglia are activated in response to treatment with rotenone; this was in agreement with other studies using animal models [2,12,22,23]. Interestingly, and in contrast to dopaminergic SH-SY5Y cells, CHME-5 cells do not show the formation of inclusions/aggregates at the end of week 4. The presence/absence of inclusion bodies was confirmed using both H & E staining to identify any eosinophilic aggregates and immunocytochemistry to detect any α-synuclein and ubiquitin positive aggregates. FLICA assay detects apoptosis in the very early stages if cells are committed to cell death. We did not find any difference in the level of apoptosis between rotenone treated and untreated CHME-5 cells in contrast to the levels of apoptosis in SH-SY5Y cells suggesting an increased level of tolerance in the CHME-5 cells for chronic low dose rotenone. The main components identified in the inclusion bodies include α-synuclein and ubiquitin [12,16,21]. Although microglia have been shown to express both α-synuclein [30] and ubiquitin [31], the results of this study indicated that microglia do not form intracellular α-synuclein and ubiquitin positive, eosinophilic protein aggregates, and are resistant to apoptosis and damage induced by rotenone. Understanding the mechanisms by which microglia are protected from the toxic effects of rotenone as well as pathways/factors by which microglia accelerate neuronal death is the next important step.

Studies have shown that rotenone treatment induces mitochondrial impairment leading to excessive generation and accumulation of ROS (Reactive Oxygen Species) [12,15,21,32] and that intracellular ROS can accelerate protein cross-linking within cells [21,29]. We have previously shown a 48% increase in the intracellular ROS in SH-SY5Y cells within one week of treatment with rotenone [21]. These cells showed protein aggregates and intracellular inclusions within four weeks, with the accumulation of ROS preceding the process of inclusion body formation in these cells [21]. Surprisingly, in the present study, we did not find any difference in the accumulation of intracellular ROS in rotenone treated CHME-5 cells compared to controls. An interesting observation of the present study was that the rotenone treated SH-SY5Y cells accumulate large amounts of ROS intracellularly and do not release significant amounts into the extracellular environment. However rotenone treated CHME-5 cells do not accumulate substantial amounts of ROS within cells but release large amounts of ROS into the extracellular environment (medium). This may well be one of the reasons why these human microglia do not form protein aggregates, and in addition this release of extracellular ROS may cause damage to neurons.

The major ROS generated by rotenone treatment is superoxide; superoxide dismutase (SOD) is an anti-oxidant and superoxide scavenger enzyme known to protect cells against the toxicity of superoxide [33]. There are two types of SOD: copper/zinc (Cu/Zn) SOD and manganese (Mn) SOD. Each type of SOD plays a different role in maintaing the health of cells. Cu/Zn SOD (SOD1) protects the cell's cytoplasm, while Mn SOD (SOD2) protects mitochondria from free radical damage [33,34]. We speculated that there would be a difference in the levels of SODs in the two types of cells used in this study and that this might be responsible for the different levels of ROS observed. We therefore investigated both SOD1 and SOD2 in SH-SY5Y and CHME-5 cells after treatment with rotenone. Interestingly the basal levels of both SOD1 and SOD2 were higher in SH-SY5Y cells compared to CHME-5 cells. SOD1 levels in CHME-5 cells increased approximately two fold upon exposure to rotenone, however the increase in SOD1 in SH-SY5Y cells was more dramatic. These results suggested that microglia do not accumulate ROS intracellularly and therefore the increase in SOD1 was significant but not dramatic. SH-SY5Y cells show abundant amounts of intracellular accumulation of ROS within cells [21]; therefore there is an increase in both SOD1 and SOD2 in these cells. However the dramatic increase in SOD1 affords protection against large amounts of intracellular ROS.

Microglia express genes encoding all components of phaogocytic and non phagocytic NADPH oxidases (Noxs); NOX1, P47 phox, P40 phox, P67 phox, NOXO1, NoxA1 and Rac1 and a transmembrane flavocytochrome heterodimer consisting of P22 ^phox ^and the gp91^phox ^(Nox2) catalytic subunit [33,35,36]. Nox2 generates superoxide ions and both Nox1 and 2 are important in the generation of cytotoxic nitrite species [37]. Several studies have reported that activated microglia generate reactive oxygen and nitrogen species [36,38]. In brains from Alzhiemers disease patients, cytocolic Nox components are observed to be markedly translocated to the membranes of microglial cells [39]. In PD brains, Nox2 has been shown to be increased in microglial cells [40] and in Central Nervous System (CNS) Nox has been shown to be predominanatly present in microglial cells when compared to other cell types [41]. Surprisingly we did not observe the intracellular accumulation of ROS (mainly superoxide) within microglia. Cheret et al, [35] observed intracellular accumulation of superoxide only in Zymosan engulfing microglia and only within phagosomes. We did not use zymosan or any other agent that microglia can engulf, so this may explain in part why we did not see intracellular ROS accumulation. The results from Cheret et al, [35] and from our study suggest that ROS accumulate in microglia only when they are engulfing an agent and ROS are generally compartmentalised within phagosomes.

Some researchers believe that superoxide diffusion through the phospholipid bilayer is poor and that cytoplasmic superoxide anions do not cause neurotoxicity directly [33,42]. However Gao et al, [22,23] and others [43] have attributed rotenone induced neurotoxicity to the release of NADPH oxidase derived superoxide. The main question that remains is that if superoxide ions do not diffuse across the microglial membrane, then how do microglia accelerate rotenone induced neurotoxicity? It has been suggested that superoxide is converted into membrane permeable hydrogen peroxide (H_2_O_2_) and other down stream products such as single oxygen species and very aggressive hydroxyl radicals (OH^-^); these products are released into the extracellular environment and can cross the neuronal membranes or can cause direct neurotoxicity. In our study, we have observed excessive amounts of ROS in the extracellular culture medium of the rotenone treated CHME-5 cells. Dopaminergic neurons are inherently prone to oxidative stress due to their characteristics of accumulation of high amounts of iron and dopamine [44,45]. In the presence of redox active iron, highly reactive and toxic hydroxyl radicals (OH^-^) are generated from H_2_O_2 _and superoxide [46]. Most importantly, extracellular ROS appear to promote mitochondrial generation of intracellular ROS in dopaminergic cells [47]. Therefore microglia originated superoxide induced by rotenone might damage dopaminergic neurons directly via formation of membrane permeable downstream products or may promote neuronal mitochondrial ROS production that is a result of complex I enzyme inhibition.

Different treatments and signalling pathways lead to the production of either superoxide or nitric oxide (NO) using Nox or iNOS pathways respectively [33]. Since human microglia produce little or no iNOS [24,25], the main pathway in human microglia for superoxide production appears to be via Nox. Activation of the Nox system results in the generation of superoxide within minutes [36]. While the ROS generated by microglial cells have the potential to harm the producing cells themselves, these do not appear to cause substantial damage to microglial cells [33], suggesting that microglial cells are well equipped with a strong antioxidative defense mechanisms to protect against oxidative and nitrosative damage. The first line of defence against ROS is the small molecular weight components such as glutathione, ascorbate and α-tocopherol. These compounds react easily with radicals [33,48], and additionally, the antioxidant enzymes such as SODs, catalase, glutathione peroxidase (Gpx) and glutathione reductase (GR) also play an important role in clearing the ROS [33]. Microglia have been shown to contain the highest levels of glutathione (GSH) compared to astrocytes and oligodendrocytes in rat brain cell cultures and in the rat mixed primary cell cultures [49,50]. Depending on the type of stimulus, the microglial content of GSH can be increased by increased GSH synthesis or decreased by increased consumption of GSH [49,51]. In addition, the presence of several antioxidant enzymes such as SOD1, GPx, and catalase has been reported in brain sections of rat and human origin. Interestingly MnSOD (SOD2) was not detected in unstressed conditions [33,34,52] and peroxyredoxin 1, an antioxidant protein was observed only in activated microglia [53]. It is thought that as soon as superoxide is produced by Nox, it is quickly converted to O_2 _and H_2_O_2 _in microglia by SODs. H_2_O_2 _reacts with GSH or with NO to form peroxynitrite. H_2_O_2 _and peroxynitirite are membrane permeable and can directly damage neurons. In addition H_2_O_2 _and ROS also facilitate phagocytosis and stimulate expression of microglial Tumour Necrosis Factor-α (TNF-α) [54]. In relation to anti-oxidative mechanisms, it could be said that microglial cells are well equipped with a strong GSH system and other anti-oxidative enzymes and can therefore adapt easily to stress conditions.

## Conclusion

In Conclusion, we have shown that similar to microglia of animal origin, human microglia (cell line) are activated in response to chronic low dose rotenone treatment. Human microglia do not show accumulation of excess amounts of intracellular ROS, however release abundant amounts of ROS into the extracellular environment (culture medium). Chronic low dose rotenone treatment does not induce accelerated apoptosis in microglia. Although human dopaminergic SH-SY5Y neurons show excessive amounts of both SODs, neurons develop eosinophilic inclusion bodies positive for both α-synuclein and ubiquitin within four weeks of rotenone treatment. The elevated levels of SODs in neurons appear to be in response to high levels of ROS generated within the neurons themselves upon rotenone treatment. Microglia appear to be 'resistant' to rotenone (chronic low dose) induced toxicity and do not show protein aggregation or inclusion body formation. Although the mechanism of microglial defence/resistance against rotenone toxicity can be explained in part by our results and the available current literature, further in depth studies need to be undertaken to fully explain this resilience.

## Methods

### Cell Culture and Rotenone Treatment

We used the transformed human microglial cell line CHME-5 in this study. The availability of human tissue, low yield of human microglia and ethical issues associated with the use of the human tissue pose significant challenges for establishing primary human microglia. CHME-5 (a generous gift from Professor Pierre Talbot, Laboratory of Neuroimmunovirology, INRS-Institute, Armand-Frappier, Canada) was obtained from embryonic fetal human microglia through transformation with SV-40 virus [55,56]. This cell line has been well characterized by Prof Talbot's group and others [55-57]. These cells express the antigens present on the adult human microglia, secrete the pro-inflammatory cytokines upon activation, exhibit the properties of primary human microglia and have been successfully used as a model for microglial activation by others [55,57,58]. Cells (1.7 × 10^2 ^cells/25 cm^2^) were grown in complete DMEM-F12 (Dulbecco's Modified Eagle Medium-F12) medium with 10% FBS (Fetal Bovine Serum), 2 mM glutamine and 1 mM sodium pyruvate at 37°C under 5% CO_2 _in air. Media were supplemented with 5 nM rotenone (Sigma Aldrich, USA) or with solvent (DMSO, used to dissolve rotenone) for 4 weeks. The concentration of rotenone was based on our previous work [21] where we have shown that treatment of human dopaminergic cells (SH-SY5Y) with 5 nM rotenone results in the appearance of intracellular inclusion bodies in these cells within four weeks. Cells were grown in 100 mm plates and fed twice weekly with the respective/appropriate media. Cultures were passaged on reaching confluence. Cover slips of a 12 mm diameter were placed in each plate. Three/four coverslips were retrieved from each treatment group at the end of every week. Cells on coverslips, collected for immunocytochemical assays, were fixed immediately in 4% buffered paraformaldehyde for 15-20 min, washed with PBS and stored in PBS with 0.1% sodium azide at 4°C until further use. Cells and cell lysates collected at the end of week 4 were used for immunocytochemical (for α-synuclein, ubiquitin and CR/43 proteins), biochemical (FLICA-apoptosis assay and DHE-ROS assay), Haematoxylin-Eosin (H & E) staining of cells and western blot assays (for Glut-5 and SODs levels). Extracellular ROS released by cells into the culture media were measured at week 4 using Acridan Lumigen PS-3 assay.

### Detection of intracellular inclusion bodies by H & E and Immunofluorescent staining

#### a. Haematoxylin-Eosin (H & E) staining

To confirm the presence/absence of inclusion bodies in the CHME-5 cells, cells grown on coverslips and paraformaldehyde (PFA) fixed were stained with H & E using a standard protocol. Briefly, cells were incubated with 2% Gill's Haematoxylin solution for 5 minutes, rinsed with distilled water followed by 10 dips (30 sec each) in blueing Solution (supernatant of the saturated solution of Lithium chloride), washed with water for 5 minutes, then the cells were incubated in 1% Eosin solution for 2 minutes. Coverslips were mounted using Aquatex mount (Merck, Germany) after the final rinse in water. Inclusion bodies, known to be eosinophilic, were identified using a Leica DMR upright light microscope.

#### b. Immunofluorescent staining

α-synuclein and ubiquitin are main components of the lewy inclusion bodies within neurons; microglia also express α-synuclein [30] and ubiquitin [31]. These two proteins were therefore used as markers to monitor the development of intracellular inclusion bodies within microglial cells. Cells grown on cover slips were processed for the detection of α-synuclein positive inclusion bodies as previously described [21]. Briefly, cells collected at week 4 were processed for the detection of α-synuclein using immunofluorescent (immunocytochemical) techniques. The coverslips were placed in 24 well plates, cells were blocked for non-specific reactivity by incubating cells/coverslips with 20% normal goat serum in PBS-T for 2 hrs, washed three times with PBS and then incubated with rabbit anti-human α-synuclein (Chemicon International Inc, USA; 1:500 diluted in the blocking solution) for 3 hrs followed by 2 hrs incubation with anti-rabbit Alexa 568 (red; Molecular Probes, USA) secondary antibody at 1:400 dilution in the blocking solution. For ubiquitin staining, cells were blocked with 10% normal goat serum in PBS-T for 2 hrs, followed by incubation with rabbit anti-ubiquitin antibody (Dako, USA, 1:1000 diluted in the blocking solution) for 3 hrs and anti-rabbit Alexa 488 secondary antibody (green; Molecular Probes, USA, 1:500 diluted in the blocking solution). Hoechst 33258 (Molecular Probes, USA) was used to stain the cell nucleus. Coverslips were mounted using prolong gold mounting medium (Molecular Probes, USA). Images were recorded using a Leica DMR fluorescent microscope.

Since we have already shown that rotenone treatment induces inclusion body formation in SH-SY5Y human dopaminergic cells within 4 weeks [21], we used these cells as a positive control for inclusion body formation in CHME-5 cells. These cells were treated similarly as described previously [21] before being processed for H & E, α-synuclein and ubiquitin staining using the specific antibodies described above.

### Detection of ROS (Reactive Oxygen Species)

#### a. Intracellular ROS

ROS (mainly superoxide) generated within the cells were detected using dihydroethidium (DHE; Molecular Probes, Invitrogen, USA) as described previously [21]. DHE is a cell permeable fluorescent dye that is oxidized by ROS present in the cytoplasm, and in an oxidized state, intercalates with nuclear DNA and is detectable as red fluorescence [21,59]. The extent of DHE fluorescence corresponds to the amount of ROS present in cells. Both cell types (CHME-5 and SH-SY5Y) cultured on coverslips from two treatment groups as described above (DMSO control and 5 nM rotenone) were collected in 24 well plates at the end of week 4 (28 days), the cells incubated with 5 μM DHE (diluted in the respective media) at 37°C for 30 minutes. After incubation, cells were washed in PBS and fixed in 4% buffered paraformaldehyde for 10 minutes, washed with PBS and treated with Hoechst 33258 nuclear dye (Molecular Probes, Invitrogen, USA; 1:500 diluted in PBS) for 5 minutes. Cells were washed again with PBS and coverslips mounted with Prolong gold mounting medium (Molecular Probes, USA). All images (2560 × 1920 pixels) were collected at the same camera settings using a Leica DMR Fluorescent microscope. DHE fluorescence was analyzed using Image J software. Since the area of interest was the nucleus of each cell, a boundary was drawn around each nucleus (using RGB images) and the marked areas saved in ROI (Region of Interest) manager. The ROI were opened as grey scale images and the mean fluorescence intensity of pixels (mean grey value) within the nuclear boundaries determined for all the images. 50 cells/treatment group/cell type were used for ROS measurements.

#### b. Extracellular ROS

ROS released into the medium by the cells were measured in the culture media collected at the end of the week 4 using the Acridan Lumigen PS-3 assay (developed in our laboratory) using a 96 well plate format. Acridan compounds (or acridinium derivatives) can be oxidised enzymatically or electrochemically; in both cases oxidation products generate chemiluminescence [60,61]. Acridan compounds are therefore widely used as a chemiluminescent substrate for the detection of bands using enzymatic oxidation in a western blot method. We used Acridan Lumigen PS-3, provided by GE Healthcare (UK), as a chemiluminescent substrate for HRP (Horse Radish Peroxidase) in the ECL plus kit. Lumigen PS-3 reacts with hydrogen peroxide and forms acridinium esters that can be oxidised with free radicals and excited products emit light at 430 nm. For the purpose of measuring ROS in the culture media, we mixed Reagent A (H_2_O_2_, ECL plus kit, GE Healthcare, UK) and Reagent B (Acridan substrate, ECL plus kit, GE Healthcare, UK) as per the manufacturer's instructions (in 40:1 ratio); this mixture was named the PS-3 substrate. 50 μl of each sample from three different replicates were plated in a 96 well plate, 50 μl of TBS (Tris Buffered Saline) was added into each well followed by 50 μl of the PS-3 substrate. The plate was incubated in the dark for 2 minutes at room temperature. Chemiluminescence was recorded digitally using Fujifilm Luminescent Image Analyzer (LAS-1000, Version 1.0.0). The Light intensity (photons) was also measured using BioTek Synergy 2 plate reader and Gen5 software. The readings recorded by the Biotek plate reader were used for analysis. The specificity of the Acridan PS-3 assay in generating ROS specific chemiluminescence was confirmed in this study by using two different antioxidants. The culture media collected from rotenone treated CHME-5 cells at the end of week 4 were incubated with either 100 μM ascorbic acid (vitamin C, antioxidant) or 1:500 diluted NuPAGE antioxidant (Invitrogen) containing Sodium bisulfite, a strong antioxidant against a wide range of ROS including superoxide anions [62]. The dose of antioxidants was decided by performing pilot dose response experiments with these antioxidants (data not shown). The incubation of the media with these antioxidants was carried out for 5 minutes before adding the PS-3 substrate; chemiluminescence was recorded as described above.

### Detection of early signs of apoptosis using FLICA apoptosis kit

Apoptosis/programmed cell death is a multi-step process [63]. These stages include an *initiation stage *- cell obtains the initial cell death activation signals; *a commitment stage *- the cell becomes committed to apoptosis; *an amplification stage*: multiple caspases are activated, and *a demolition stage *- caspase mediated destruction of the cellular structure. The FLICA (Fluorochrome Inhibitor of Caspases) apoptosis detection kit is designed to detect active caspases. The Fluorochrome inhibitor (SR-VAD-FMK, Sulforhodaminyl-L-valylalanylaspartyl fluoromethyl ketone) used in this assay is cell permeable and non cytotoxic, and once in the cell, binds covalently to all active caspases. Covalently bound inhibitors are retained in the cell while any unbound reagent is washed away. Thus the remaining cell fluorescent signal is a direct measure of the amount of active caspase enzymes present at the time the reagent was added. The cells can be analysed by 96 well plate fluorometry or by fluorescence microscopy. For this study, the cells (both CHME-5 and SH-SY5Y) were collected at the end of 28 days (week 4) and FLICA assay was performed on the trypsinised cells suspended in 96 well plates, according to the manufacturer's instructions (Immunocytochemistry Technologies, LLC, Bloomington, MN). The amount of caspases was analysed by recording the fluorescent signals in the 96 well plate using Biotek Synergy 2 plate reader and Gen5 software.

### Detection of microglial activation and levels of SODs using immunocytochemical and western blotting methods

#### a. Glucose transporter type 5 and CR3/43

Glucose transporter type 5 (Glut-5) is a microglia specific protein [27,64], that contributes to the kinetics of cerebral metabolism. The expression of Glut-5 is detected at all stages of microglial activation, however its expression increases with activation [26]. We therefore used Glut-5 as the marker for microglial activation. The levels of Glut-5 were measured using Western blot methods and the polyclonal Rabbit-anti-humanGlut-5 antibody (Chemicon International Inc, USA). Briefly, 15 μg of each sample was loaded into separate wells of a 4-12% gradient SDS PAGE gel (NuPAGE, Invitrogen) and run at 200 V. A nitrocellulose membrane and MOPS transfer buffer (with 10% methanol) were used for transfer at 30 V for 1 hr at 4°C. The membrane was blocked with 5% non fat milk in PBS-T (PBS with 0.1% Tween 20) for 16-18 hrs at 4°C. After blocking, the membrane was washed three times in PBS-T at room temperature (RT) followed by incubation for 3 hrs at RT with the primary antibody at 1:500 diluted in 1% milk in PBS-T. The membrane was then washed twice with PBS-T, followed by incubation with anti-rabbit-HRP conjugated secondary antibody (Sigma Aldrich, USA; 1: 16000 diluted in 1% milk in PBS-T). Chemiluminescent signals were captured using an ECL-plus chemiluminescent kit (GE Healthcare, UK) and Fujifilm Luminescent Image Analyzer (LAS-1000, Version 1.0.0).

Microglial activation was also confirmed using CR3/43 as a marker and immunocytochemical method. CR3/43 also known as HLA DR/DP/DQ belongs to the family of Human Leukocyte Antigens (HLA) with important roles in the immune system. CR3/43 protein is specifically expressed in activated microglia and is therefore used as a marker for activated microglia [65,66]. To confirm the activated state of microglia, the cells grown on colverslips were fixed in 4% buffered paraformaldehyde, washed with PBS and processed for the detection of CR3/43 protein at the end of the week 4 using the monoclonal CR3/43 antibody (abcam, UK, 1:100 diluted in the blocking solution) and respective secondary antibody (Alexa 488 conjugated, 1: 400 diluted, Molecular Probes). Cells were washed three times with PBS-T (PBS having 0.1% Tween 20) after primary and secondary antibody incubations. Following secondary antibody washes, cells were treated with Hoechst 33258 nuclear dye (Molecular Probes, Invitrogen, USA; 1:500 diluted in PBS) for 5 minutes, washed with PBS and coverslips mounted with Prolong gold mounting medium (Molecular Probes, USA). Images (2560 × 1920 pixels) were recorded using a Leica DMR Fluorescent microscope.

#### b. Superoxide dismutases (SODs)

SOD 1 and 2 were used to assess the ability of microglia to 'defend' against the accumulation/generation of Reactive Oxygen Species (ROS). Polyclonal anti-rabbit antibodies for SOD1 (abcam, UK) and SOD2 (gift from Professor Mike Berridge, Malaghan Institute of Medical Research, Victoria University, Wellington, New Zealand) were used.

The levels of SOD 1 and 2 were measured using western blot methods as described above for Glut-5 levels; the concentrations of primary antibodies were 1: 5000 and 1:1000 for SOD 1 and 2 respectively. Anti-rabbit secondary antibody was used at 1:16,000 dilution for both the targets. Chemiluminescent signals were recorded using an ECL-plus chemiluminescent kit (GE Healthcare, UK) and Fujifilm Luminescent Image Analyzer (LAS-1000, Version 1.0.0).

### Statistical analysis

Each experiment described above was repeated three times and data from three independent experiments used for statistical analysis. Differences between the two groups were compared using a Student's t test (Prism software, version 3.02, GrapPad Inc, USA) with P < 0.05 considered a significant difference. Results shown are representative of three independent experiments. All data were expressed as the mean ± standard deviation.

## Competing interests

The authors declare that they have no competing interests.

## Authors' contributions

SBS conceived of the study, carried out experimental work, analysed and interpreted results and prepared the manuscript. LFBN critically reviewed the manuscript. Both authors have read and approved the final manuscript.
